# Supplementary material for: TRIAC Treatment Improves Impaired Brain Network Function and White Matter Loss in Thyroid Hormone Transporter Mct8/Oatp1c1 Deficient Mice
Source: Int J Mol Sci. 2022 Dec 8;23(24):15547. doi: 10.3390/ijms232415547 (PMC9779161; doi:10.3390/ijms232415547)
Supplement: Supplementary file 1 [file ijms-23-15547-s001.zip › ijms-2043375-supplementary.pdf]

Supplemental Information

TRIAC treatment improves impaired brain network function and white matter loss in thyroid hormone transporter Mct8/Oatp1c1 deficient mice

Jonathan Rochus Reinwald, Wolfgang Weber-Fahr, Alejandro Cosa Linan, Robert Becker, Markus Sack, Claudia Falfan-Melgoza, Natalia Gass, Urs Braun, Christian Clemm von Hohenberg, Jiesi Chen, Steffen Mayerl, Thomas F. Muente, Heike Heuer, Alexander Sartorius

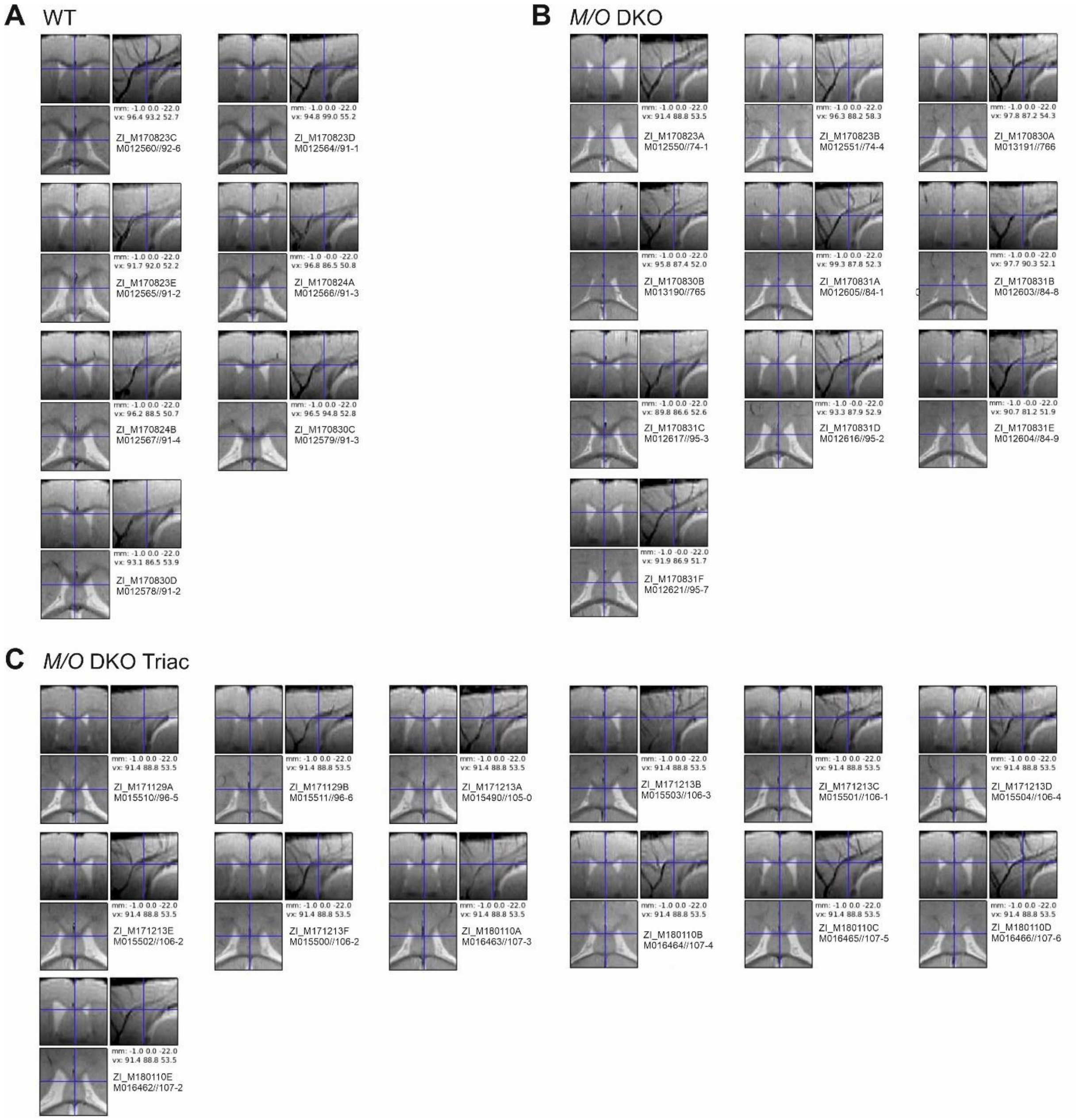

Figure S1: **Illustration of white matter volume differences between WT, *Mct8/Oatp1c1* DKO and *Mct8/Oatp1c1* DKO TRIAC mice on single animal level**.

Single animal structural magnetic resonance images with slices focusing on white matter of the corpus callosum are illustrated for **(A)** WT mice (n=7), **(B)** *Mct8/Oatp1c1* DKO (n=10) and **(C)** *Mct8/Oatp1c1* DKO mice treated with TRIAC (n=13). Images have been co-registered and normalized but otherwise not being preprocessed. While prominent white matter in the corpus callosum is visible in **(A)**, *Mct8/Oatp1c1* DKO mice in **(B)** show strong reduction of white matter volume, and TRIAC treated *Mct8/Oatp1c1* DKO mice demonstrate a partial normalization of the white matter thinning **(C)**.

WT, wild type; *M/O* DKO, *Mct-8/Oatp1c1* double knockout; *M/O* DKO TRIAC, *Mct-8/Oatp1c1* double knockout mice treated with triiodothyronine during the first three postnatal weeks.

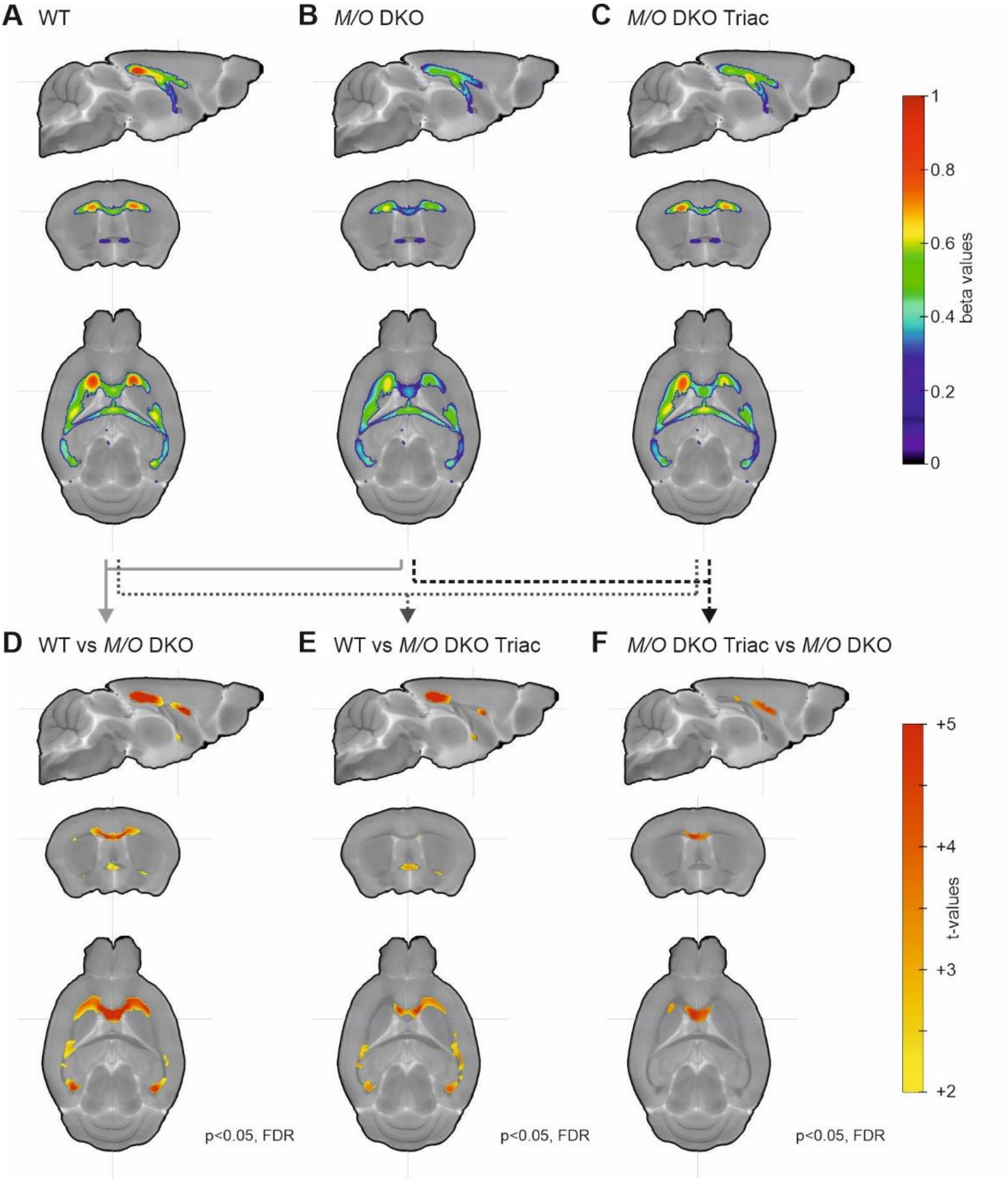

Figure S2: **White matter volume differences between WT, *Mct8/Oatp1c1* DKO and *Mct8/Oatp1c1* DKO TRIAC mice**.

(A-C) White matter beta maps obtained from SPM12 second-level analysis (GLM) with the three groups as regressors of interest, age and total intracranial volume as covariates, and the modulated warped images as input. **(D-F)** quantify the differences in white matter volume between the three groups demonstrating significantly larger white matter volume in WT compared to *Mct8/Oatp1c1* DKO mice **(A)**, while differences to *Mct8/Oatp1c1* DKO TRIAC mice were less pronounced, albeit still existent **(B)**. Together with the higher white matter volume in *Mct8/Oatp1c1* DKO mice treated with TRIAC in comparison to the untreated group **(F)**, our results suggest a partial but not full recovery effect by the treatment with TRIAC, which becomes also obvious in the comparison between the beta maps **(B and C)**. White matter volumes were corrected for age and overall brain volume as covariates.

WT, wild type; *M/O* DKO, *Mct-8/Oatp1c1* double knockout; *M/O* DKO TRIAC, *Mct-8/Oatp1c1* double knockout mice treated with triiodothyronine during the first three postnatal weeks; FDR, false discovery error rate correction.

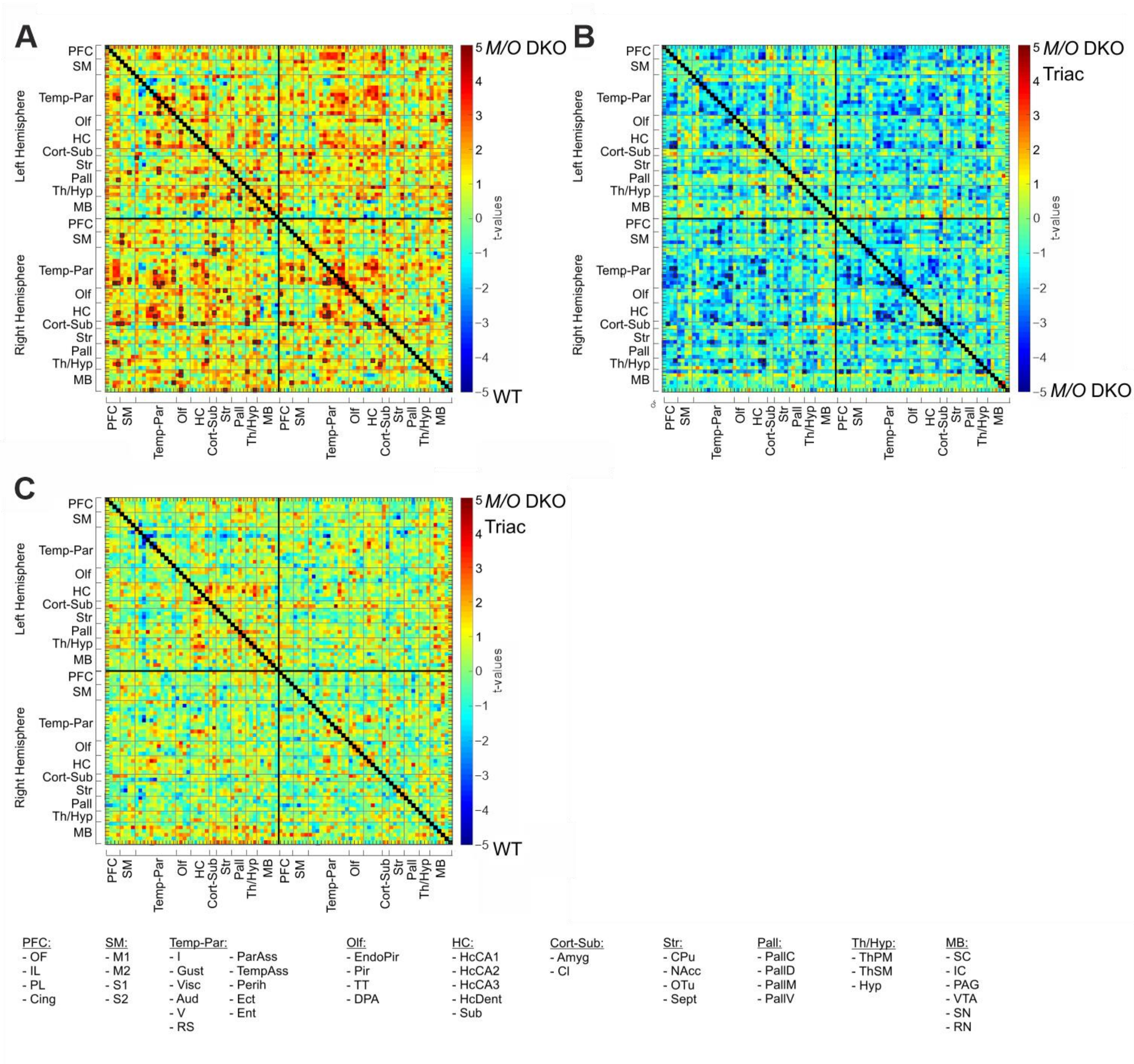

Figure S3: **Differences in functional connectivity between WT, *Mct8/Oatp1c1* DKO and *Mct8/Oatp1c1* DKO TRIAC mice**.

Network-based statistic (NBS) revealed significantly higher functional connectivity between multiple brain regions in *Mct8/Oatp1c1* DKO compared to WT (**A**, orange to red connections marked with black boxes). Early-life treatment of *Mct8/Oatp1c1* DKO mice with TRIAC normalized this hyperconnectivity, demonstrating on the one hand a significantly lower connectivity compared to the untreated *Mct8/Oatp1c1* DKO mice (**B**, light to dark blue connections marked with black boxes) within a pattern covering almost the same regions as in **A** and on the other hand no significant group differences compared to WT mice (**C**). The schemaball **(D)** illustrates the overlapping connections between (**A**) and (**C**), representing the recovery effect of the treatment with TRIAC in the *Mct8/Oatp1c1* DKO mice. NBS results are calculated on functional connectivity matrices adjusted for age as a covariate with primary thresholds between $t_{(15)}>3.73$ (**A**), $t_{(21)}>3.53$ (**B**), and $t_{(18)}>3.62$ and a p-value for the permutation test (5.000 permutations) of $p_{NBS}<0.025$.

WT, wild type; *M/O* DKO, *Mct8/Oatp1c1* double knockout; *M/O* DKO TRIAC, *Mct8/Oatp1c1* double knockout mice treated with *TRIAC during the first three postnatal weeks.* For brain regions abbreviations see Figure 1.

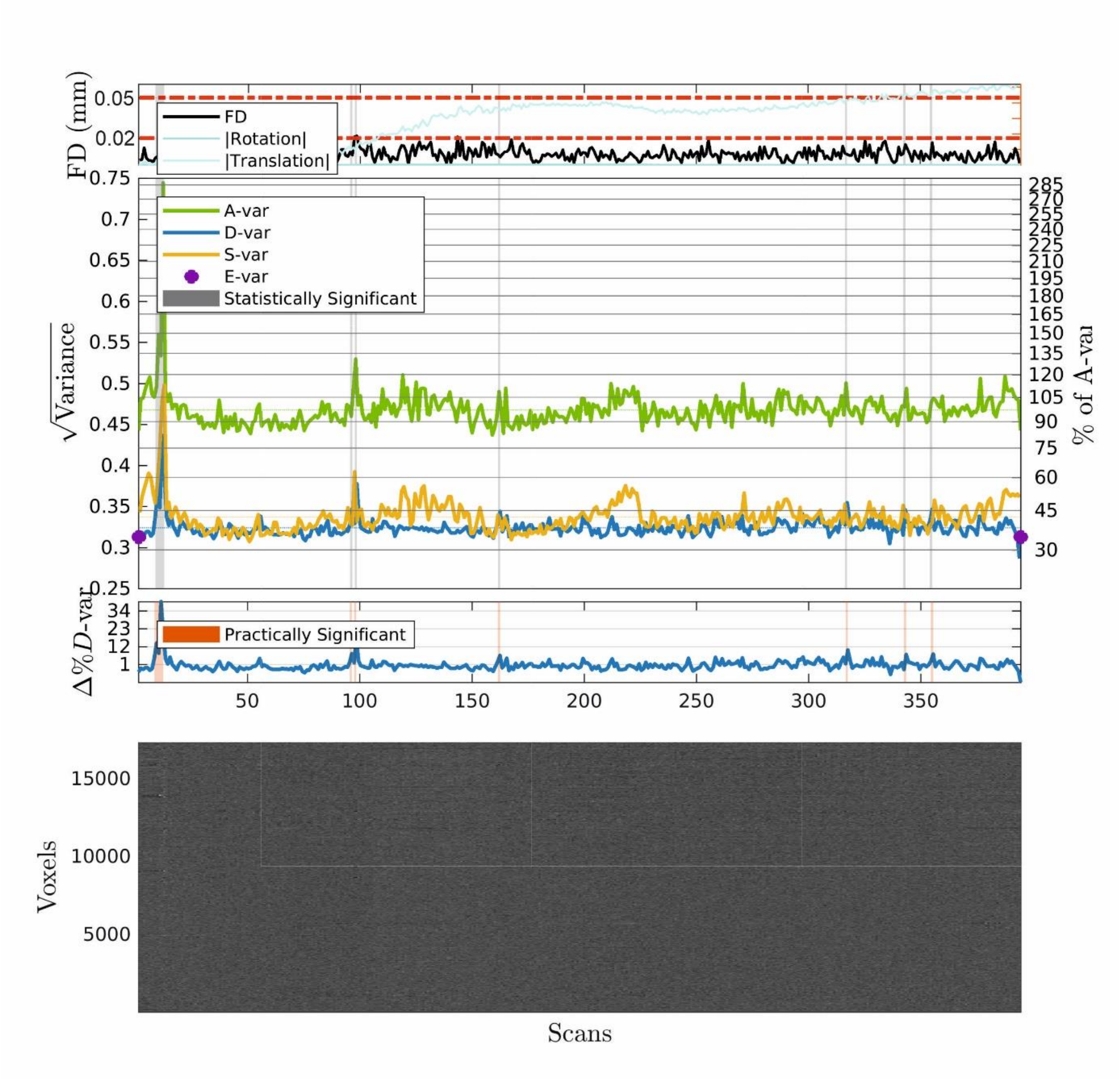

Figure S4: **DSE and DVARS inference for pre-processed data of an exemplarily (typical) animal based on [1].**

The upper panel shows four plots, framewise displacement (FD), the DSE plot, the percentual delta-value of D-Var in percent, and an image of all brainordinate elements. FD plots show 0.02 mm and 0.05 mm, strict and lenient thresholds, respectively. All time series plots have DVARS test significant scans marked, gray if only statistically significant (5% Bonferroni), in orange if also practically significant (Δ%D-var>5%). Note the motion artefact around frame 10.

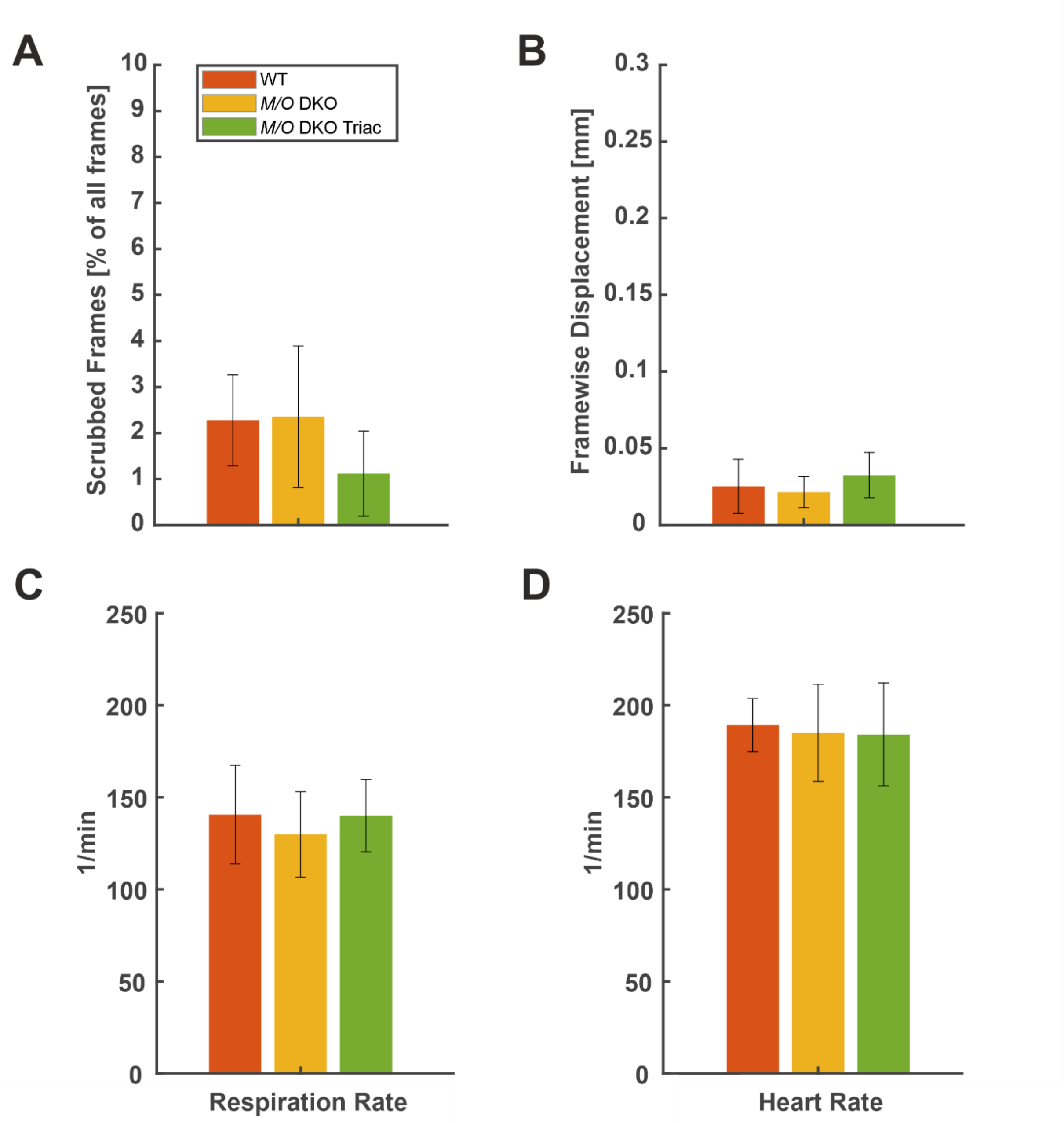

Figure S5. **Number of scrubbed frames, framewise displacement and physiological parameters for WT, *Mct8/Oatp1c1* DKO and *Mct8/Oatp1c1* DKO TRIAC mice**.

(A) Mean values (+/- SEM) of scrubbed frames. **(B)** Mean values (+/- SEM) of framewise displacement. **(C)** Mean values (+/- SEM) of respiration rate (mean per animal). **(D)** Mean values (+/- SEM) of heart rate (mean per animal). Results from one-way ANOVA show no significant difference ($p>0.05$) for **(A-D)**.

WT, wild type; *M/O* DKO, *Mct-8/Oatp1c1* double knockout; *M/O* DKO TRIAC, *Mct-8/Oatp1c1* double knockout mice treated with triiodothyronine during the first three postnatal weeks.

Literature

1. Afyouni, S. and T.E. Nichols, *Insight and inference for DVARS.* Neuroimage, 2018. **172**: p. 291-312.
